# Replication of recently identified associated single-nucleotide polymorphisms from six autoimmune diseases in Genetic Analysis Workshop 16 rheumatoid arthritis data

**DOI:** 10.1186/1753-6561-3-s7-s31

**Published:** 2009-12-15

**Authors:** Harshal Deshmukh, Xana Kim-Howard, Swapan K Nath

**Affiliations:** 1 Genetic Epidemiology Unit, Arthritis and Immunology Research Program, Oklahoma Medical Research Foundation, 825 Northeast 13th Street, Oklahoma City, Oklahoma 73104 USA

## Abstract

Many autoimmune diseases share similar underlying pathology and have a tendency to cluster within families, giving rise to the concept of shared susceptibility genes among them. In the Genetic Analysis Workshop 16 rheumatoid arthritis (RA) data we sought to replicate the genetic association between single-nucleotide polymorphisms (SNPs) identified in recent genome-wide association studies (GWAS) on RA and five other autoimmune diseases. We identified 164 significantly associated non-HLA SNPs (*p *< 10^-5^) from 16 GWAS and 13 candidate gene studies on six different autoimmune diseases, including RA, systemic lupus erythematosus, type 1 diabetes, Crohn disease, multiple sclerosis, and celiac disease. Using both direct and imputation-based association test, we replicated 16 shared susceptibility regions involving RA and at least one of the other autoimmune diseases. We also identified hidden population structure within cases and controls in Genetic Analysis Workshop 16 RA data and assessed the effect of population structure on the shared autoimmunity regions. Because multiple autoimmune diseases share common genetic origin, these could be areas of immense interest for further genetic and clinical association studies.

## Background

Autoimmune diseases affect 5% of the human population [1]. Although there is considerable heterogeneity among these disorders, their manifestations are believed to arise from immune-mediated attack against self-antigens. Despite their clinical heterogeneity, recent studies examining gene expression profiles in peripheral blood mononuclear cells (PBMC) of individuals with autoimmune disorders reveal common features that are either shared within a disease group or among disease groups as exemplified in rheumatoid arthritis (RA) [2] or in systemic lupus erythematosus (SLE) [3]. The major symptoms of RA arise through immune-mediated destruction of peripheral joints; however, these features are typically accompanied by systemic complications such as rheumatoid nodules and vasculitis. Immune-mediated destruction is the central feature of autoimmune diseases like SLE, type 1 diabetes (T1D), multiple sclerosis (MS), and celiac disease (CLD). Given the similarities in the basic pathology of these autoimmune disorders, it is not surprising to see autoimmune diseases clustering within families, which leads to the hypothesis of common autoimmunity genes being shared between diseases. An example of such shared gene is *Runx1*, which is shown to be associated with SLE, psoriasis, and RA [4]. Increasing numbers of GWAS for autoimmune disorders have enhanced the possibility of identifying such shared autoimmune regions.

The goals of the present study are 1) to identify population structure in Genetic Analysis Workshop (GAW) 16 RA cases and controls, 2) to replicate the genetic association in RA identified from recent GWAS on six common autoimmune diseases [RA, Crohn disease (CD), CLD, SLE, MS, and T1D], and 3) to study the effect of admixture on associated regions.

## Methods

After searching the PubMed database we identified recently published 16 GWAS and other 13 candidate gene association studies [5-28] on RA, CD, SLE, MS, CLD, and T1D. SNPs which showed significant association at a genome-wide "suggestive" threshold (*p *< 10^-5^) were chosen for replication in GAW16 RA data. The preselected threshold (*p *< 10^-5^) was chosen as "suggestive" to control properly the family-wide type 1 error as recommended by Duggal et al. [29] to adjust *p*-value to control the family-wide type 1 error in genome-wide association studies. The rationale for choosing this threshold was to maximize true associations from the GWAS. We performed an association analysis using predefined quality control criteria (MAF ≥ 1%, SNP missingness rate of ≤ 10%, and Hardy-Weinberg equilibrium ≥ 0.001 in controls) and identified significant SNPs for RA either by direct association using PLINK [30] or by imputation using fastPHASE [31].

To identify the hidden population structure in cases and controls, we estimated and compared the likelihood of this data under different numbers of ancestral populations (*k*). We used STRUCTURE [32] for estimating the best *k *separately for cases and controls. We identified 343 ancestry informative markers (AIMs) from two previously published reports [33,34] that were available in GAW16 RA data. These AIMs were used in both estimating population structure and admixture proportion in each individual, as well as correcting for the effect of population substructure in genetic association. We employed two different methods for controlling the effect of population substructure, i.e., structured association test (SAT) [35] with 10,000 permutations and covariate-adjusted logistic regression. We also included sex as a covariate in the logistic regression model; however, it did not significantly affect the association results and was excluded from the final model. To corroborate the evidence of population structure we performed principal-component analysis using EIGENSOFT. We evaluated the statistical significance of each eigenvector using Tracy-Widom (TW) statistics as described by Patterson et al. and calculated the total variation explained by the significant eigenvector [36].

Finally, we sought to replicate regions that showed association signals across GAW16 data and at least one of the GWAS. If the associated SNPs were not present (either failed or were not genotyped in the study) in the GAW16 data, we looked at the surrounding region in the GAW data (100-kb region centered on the published associated SNP). If any of the SNPs from these regions showed significance at a replication threshold of *p *< 0.05, we imputed this region using HAPMAP data (60 unrelated CEU parents) and assessed association.

## Results

**Figure 1:**
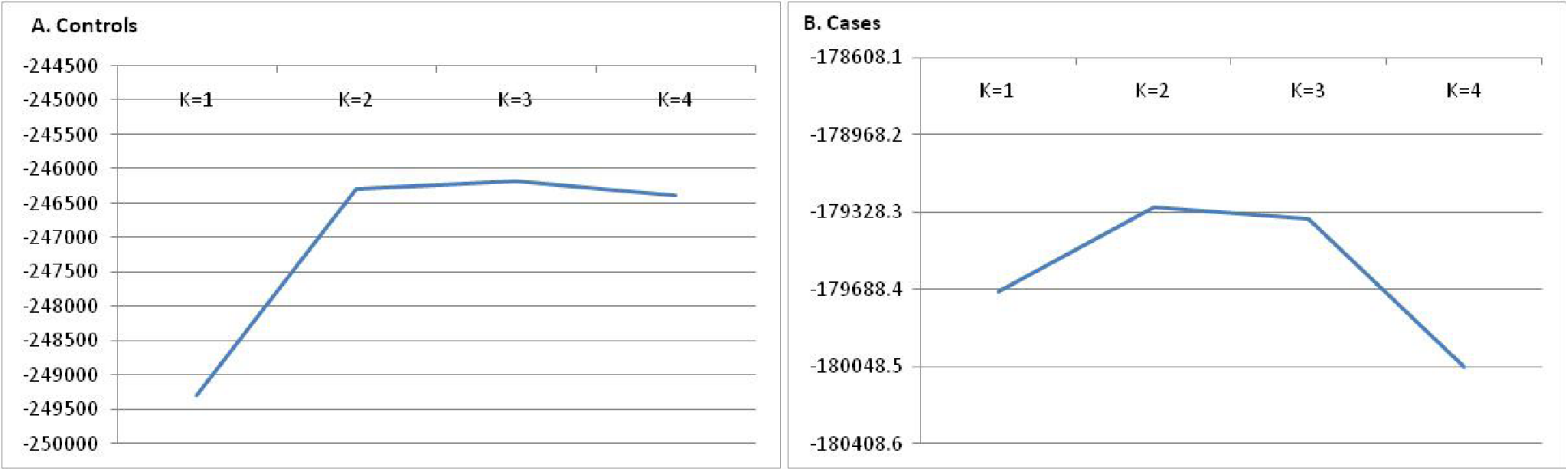
**Likelihood of data under number of hidden populations (K) estimated separately for controls (A) and cases (B)**. K denotes number of populations.

We have identified substantial population substructure in GAW16 RA samples. Figure 1A and 1B show estimated structured likelihood probability of data for cases and controls, respectively. The best fitted model for cases favored the assumption of a two-population model (ancestry proportion = 0.955, 0.045) and three-population model for controls (ancestry proportion = 0.771, 0.115, 0.074). However, a combined case-control data favored a three-population model (ancestry proportion = 0.528, 0.257, 0.215). For controls, the likelihood probabilities for two-, three-, and four-population models are similar and that for cases, the likelihood probabilities for a two- and three-population model is similar. We ran principal-components analysis on the combined cases-control data and calculated TW statistics [36] for the top 10 eigenvectors, and 4 significant eigenvectors (*p *> 0.05) explained 23% of the variation in the whole dataset. This suggests substantial population structure within GAW16 data.

We initially selected 164 non-HLA associated SNPs from 16 recently published GWAS and 13 candidate gene association studies (*p *< 10^-5^) to check for replication in the GAW16 dataset. We found associated SNPs for SLE (*n *= 49), CD (*n *= 39), T1D (*n *= 32), RA (*n *= 37), CLD (*n *= 4), and MS (*n *= 9). Of these 164 SNPs, 92 SNPs were found in the GAW16 data and evaluated by a direct allelic association test. The remaining 72 SNPs were assessed by indirect association (by imputation). Of these 164 SNPs, 29 were significantly replicated (*p *< 0.05). Nine of these SNPs replicated at *p*-values between 0.05 and 0.01, 11 were between 0.01 and 10^-5^, and 8 replicated at *p *< 10^-5^. Table 1 shows susceptibility loci with the *p*-values for autoimmune diseases (CD, CLD, T1D, SLE, and RA) identified from various GWAS. The last two columns show association based *p*-values for the same loci in the entire GAW16 RA data and *p*-values adjusted for population admixture.

**Table 1:** Replication of association in multiple autoimmune diseases

							Corrected *p*-value	
							
Chromosome number	Cytogenetic position	Gene	SNP	Physical position	Associated diseases	Uncorrected GAW *p*-value^a^	Adjusting with ancestry as covariate in a logistic regression model	SAT^b^
1	1p31	*IL23R*	rs11465804	67414547	CD	1.09 × 10^-3^	1.04 × 10^-3^	2.04 × 10^-3^
1	1p13	*PTPN22*	rs2476601	114089610	SLE, RA, T1D	1.12 × 10^-12^	1.76 × 10^-10^	2.66 × 10^-10^
2	2q24	*IFIH1*	rs1990760	162949558	T1D	6.54 × 10^-3^	2.74 × 10^-2^	2.44 × 10^-2^
2	2q32.2-q32.3	*STAT4*	rs6752770	191681808	RA, SLE	7.00 × 10^-3^	1.36 × 10^-2^	3.36 × 10^-2^
3	3p21	*MST1*	rs3197999	49696536	CD	2.31 × 10^-2^	3.57 × 10^-2^	3.57 × 10^-2^
4	4q27	*KIAA1109*	rs13151961	123473107	Celiac T1D, RA	4.81 × 10^-2^	2.74 × 10^-2^	3.74 × 10^-2^
5	5p13	*PTGER4*	rs4613763	40428485	CD	1.96 × 10^-3^	7.56 × 10^-3^	5.56 × 10^-3^
6	6q23	*near TNFAIP3*	rs6933404	138000928	SLE	3.13 × 10^-4^	2.01 × 10^-3^	3.01 × 10^-3^
6	6q23	*near TNFAIP3*	rs13192841	138008907	SLE	2.93 × 10^-4^	5.71 × 10^-4^	6.47 × 10^-4^
6	6q23	*near TNFAIP3*	rs12527282	138008945	SLE	2.28 × 10^-4^	3.37 × 10^-4^	2.27 × 10^-4^
6	6q23	*near TNFAIP3*	rs2327832	138014761	SLE	1.06 × 10^-4^	7.51 × 10^-4^	6.51 × 10^-4^
6	6q23	*near TNFAIP3*	rs602414	138053358	SLE	6.03 × 10^-4^	1.29 × 10^-2^	1.29 × 10^-2^
6	6q27	*CCR6*	rs2301436	167408399	CD	1.67 × 10^-2^	1.74 × 10^-2^	4.25 × 10^-2^
8	8p23.1	*XKR6*	rs11783247	10826285	SLE	4.50 × 10^-2^	1.76 × 10^-2^	5.77 × 10^-2^
8	8p21.1	*C8orf12*	rs7836059	11309574	SLE	8.87 × 10^-3^	1.36 × 10^-2^	6.78 × 10^-2^
8	8p21.3	*C8orf13-BLK*	rs2736340	11381382	SLE	1.45 × 10^-5^	2.38 × 10^-5^	0
8	8p21.3	*C8orf13-BLK*	rs13277113	11386595	SLE	3.46 × 10^-6^	5.69 × 10^-6^	0
8	8p23.1	*BLK*	rs2618476	11389950	SLE	*3.21 × 10*^-6^	4.10 × 10^-6^	* ^c^
8	8p23.1	*BLK*	rs2248932	11429059	SLE	9.79 × 10^-3^	6.49 × 10^-3^	6.69 × 10^-3^
9	*9q33.2*	*PHF19*	rs1953126	122680321	RA	2.76 × 10^-8^	4.97 × 10^-8^	0
9	9q33.2	*PHF19*	rs1609810	122682172	RA	*1.79 × 10*^-8^	3.38 × 10^-8^	*
9	9q33.2	*PHF19*	rs881375	122692719	RA	2.27 × 10^-8^	4.55 × 10^-8^	0
9	9q33.2	*PHF19*	rs6478486	122695150	RA	*1.79 × 10*^-8^	3.38 × 10^-8^	*
9	9q33.2	*near PHF19*	rs3761847	120769793	RA	1.24 × 10^-8^	3.88 × 10^-8^	0
9	9q33.2	*C5*	rs2900180	122776861	RA	6.24 × 10^-9^	1.88 × 10^-8^	0
10	10q24	*NKX2-3*	rs11190140	101281583	CD	4.93 × 10^-2^	8.10 × 10^-2^	8.80 × 10^-2^
19	19q13	*RSHL1*	rs8111071	50999246	CD	5.91 × 10^-5^	1.66 × 10^-4^	0
22	22q11.21	*UBE2L3*	rs5754217	20264229	SLE	8.94 × 10^-3^	6.34 × 10^-3^	6.57 × 10^-3^
22	22q13.2	*SCUBE1*	rs2071725	41934258	SLE	2.23 × 10^-2^	1.83 × 10^-2^	1.57 × 10^-2^

^a^Allelic association test

^b^Structured association test

^c ^*, Imputed SNP

## Discussion

There is a growing understanding that susceptibility to autoimmune diseases is due to a complex interaction of multiple genes and environmental factors, and many of these may be shared among many autoimmune diseases. In this analysis we attempted to replicate previously identified associations in multiple autoimmune diseases and inferred regions of shared autoimmunity between GAW16 data and any other autoimmune disease. We did not explore the HLA region in our study because this region has already been extensively investigated and is a very well know complex region of shared autoimmunity among various autoimmune disorders [37,38].

GWAS have emerged as an effective tool to identify common polymorphism underlying complex diseases. One of the major sources of bias in GWAS is population stratification, a variation of ancestry proportions between cases and controls. This stratification can lead to differences in allele frequency between cases and controls unrelated to disease status, consecutively leading to an increased type 1 error [9]. We used 343 AIMs and applied them to cases and controls separately to infer population structure. We have demonstrated substantial population substructure in both cases and controls. In fact, we have identified more sub-structure in controls than cases. Obviously, this would have major impact if not corrected properly while performing association studies.

We identified 16 different cytogenetic regions of shared autoimmunity between GAW16 data and at least one of the proposed autoimmune diseases. There were eight shared regions with SLE (1p13, 2q32.2-q32.3, 6p21.32, 6q23, 8p21.3, 8p23.1, 22q11.21, 22q13.2), six shared regions with CD (1p31, 3p21, 5p13, 6q27, 10q24, 19q13), four shared regions with RA (1p13, 2q32.2-q32.3, 4q27, 9q33.2), four shared regions with T1D (1p13, 2q24, 2q33, 4q27), and one shared region with CLD (4q27). Interestingly, *PTPN22 *(1p13), *STAT4 *(2q32.2-q32.3), and *KIAA1109 *(4q27) were all associated with multiple autoimmune disease. It should also be noted that SLE shared the most susceptibility genes with RA, suggesting common underlying pathologic processes perpetrated by common loci. These associations are constant, robust, and persisted after correcting for population structure. It is also noteworthy to report that none of the nine associated SNPs from MS are replicated in the GAW16 RA data.

However, our study was not an exhaustive replication with RA and the five other autoimmune diseases because SNPs were chosen using a predefined threshold (*p *< 10^-5^). It is possible that SNPs that showed weak to moderate association (0.05-10^-5^) with other autoimmune disease could have been highly associated with RA. Also, the other studies from which the list of 164 non-HLA SNPs were selected do not all control for population admixture so it is possible that we missed analyzing an important SNP in the GAW16 data. We did not evaluate that possibility. It is worth future research to look more exhaustively at SNPs found by GWAS and candidate gene analyses that do not pass genome-wide significance but are significant at the *p *< 0.05 level.

## Conclusion

It has long been suspected that autoimmune diseases may share common pathogenesis and susceptibility genes, and several recent studies [4,5] support this hypothesis. Identification of these shared regions can help in identification of novel genetic pathways in autoimmune disease causation, can increase understanding higher prevalence of different autoimmune disorders in families, and may identify targeted regions for gene therapy. Our study successfully identified 16 areas of shared susceptibility involving RA and other autoimmune diseases. These can be further explored by association and clinical studies to solve the conundrum of shared autoimmunity amongst various autoimmune diseases.

## List of abbreviations used

AIM: Ancestry informative marker; CD: Crohn disease; CLD: Celiac disease; GAW: Genetic Analysis Workshop; MS: Multiple sclerosis; PBMC: Peripheral blood mononuclear cells; RA: Rheumatoid arthritis; SAT: Structured association test; SLE: Systemic lupus erythematosus; T1D: Type 1 diabetes; TW: Tracy-Widom

## Competing interests

The authors declare that they have no competing interests.

## Authors' contributions

SKN conceived of the study, and participated in its design and coordination and helped to draft the manuscript. HAD and XK-H did the analysis and drafted the manuscript.
